# Characterization and Outcomes of SARS-CoV-2 Infection in Patients with Sarcoidosis

**DOI:** 10.3390/v13061000

**Published:** 2021-05-27

**Authors:** P. Brito-Zerón, B. Gracia-Tello, A. Robles, A. Alguacil, M. Bonet, B. De-Escalante, A. Noblejas-Mosso, R. Gómez-de-la-Torre, M. Akasbi, M. Pérez-de-Lis, R. Pérez-Alvarez, M. Ramos-Casals, on behalf of the SarcoGEAS-SEMI Registry

**Affiliations:** 1 Systemic Autoimmune Diseases Unit, Hospital CIMA-Sanitas, 08034 Barcelona, Spain; mbritozeron@gmail.com; 2 Department of Internal Medicine, Hospital Clínico, 50009 Zaragoza, Spain; bcgracia@salud.aragon.es (B.G.-T.); bescalantester@gmail.com (B.D.-E.); 3 Department of Internal Medicine, Hospital La Paz, 28046 Madrid, Spain; aroblesmarhuenda@gmail.com (A.R.); ananoblejas@lapaz.es (A.N.-M.); 4 Department of Internal Medicine, Hospital Virgen de la Salud, 45004 Toledo, Spain; almoan11@gmail.com; 5 Department of Internal Medicine, Althaia, Xarxa Assistencial de Manresa, 08243 Manresa, Spain; mariona.bonet2@gmail.com; 6 Department of Internal Medicine, Hospital Universitario Central de Asturias (HUCA), 33011 Oviedo, Spain; ricardoagdt@gmail.com; 7 Department of Internal Medicine, Hospital Infanta Leonor, 28031 Madrid, Spain; makasbi@hotmail.com; 8 Department of Anesthesiology, Complejo Hospitalario Universitario de A Coruña, 15006 A Coruña, Spain; zzizou9@hotmail.com; 9 Department of Internal Medicine, Hospital Alvaro Cunqueiro, 36213 Vigo, Spain; roberto.perez.alvarez@sergas.es; 10 Department of Autoimmune Diseases, ICMiD, Hospital Clinic, 08036 Barcelona, Spain; 11 Department of Medicine, University of Barcelona, 08036 Barcelona, Spain

**Keywords:** sarcoidosis, COVID-19, SARS-Cov-2, comorbidities, survival, hospital admission

## Abstract

To analyze the clinical characteristics and outcomes of severe acute respiratory syndrome coronavirus 2 (SARS-CoV-2) infection in patients with sarcoidosis from a large multicenter cohort from Southern Europe and to identify the risk factors associated with a more complicated infection. We searched for patients with sarcoidosis presenting with SARS-CoV-2 infection (defined according to the European Centre for Disease Prevention and Control guidelines) among those included in the SarcoGEAS Registry, a nationwide, multicenter registry of patients fulfilling the American Thoracic Society/European Respiratory Society/World Association of Sarcoidosis and Other Granulomatous Disorders 1999 classification criteria for sarcoidosis. A 2:1 age-sex-matched subset of patients with sarcoidosis without SARS-CoV-2 infection was selected as control population. Forty-five patients with SARS-CoV-2 infection were identified (28 women, mean age 55 years). Thirty-six patients presented a symptomatic SARS-CoV-2 infection and 14 were hospitalized (12 required supplemental oxygen, 2 intensive care unit admission and 1 mechanical ventilation). Four patients died due to progressive respiratory failure. Patients who required hospital admission had an older mean age (64.9 vs. 51.0 years, *p* = 0.006), a higher frequency of baseline comorbidities including cardiovascular disease (64% vs. 23%, *p* = 0.016), diabetes mellitus (43% vs. 13%, *p* = 0.049) and chronic liver/kidney diseases (36% vs. 0%, *p* = 0.002) and presented more frequently fever (79% vs. 35%, *p* = 0.011) and dyspnea (50% vs. 3%, *p* = 0.001) in comparison with patients managed at home. Age- and sex-adjusted multivariate analysis identified the age at diagnosis of SARS-Cov-2 infection as the only independent variable associated with hospitalization (adjusted *odds ratio* 1.18, 95% conficence interval 1.04–1.35). A baseline moderate/severe pulmonary impairment in function tests was associated with a higher rate of hospitalization but the difference was not statistically significant (50% vs. 23%, *p* = 0.219). A close monitoring of SARS-CoV-2 infection in elderly patients with sarcoidosis, especially in those with baseline cardiopulmonary diseases and chronic liver or renal failure, is recommended. The low frequency of severe pulmonary involvement in patients with sarcoidosis from Southern Europe may explain the weak prognostic role of baseline lung impairment in our study, in contrast to studies from other geographical areas.

## 1. Introduction

A novel coronavirus was identified in January 2020 as the etiological agent of a cluster of cases of pneumonia detected in Wuhan City (China). Severe acute respiratory syndrome coronavirus 2 (SARS-CoV-2) is the seventh coronavirus known to infect humans [1] and the lack of a prior immunity has resulted in a rapid increase in infected patients worldwide [2] with more than 110 million confirmed cases until 23 February 2021. The disease caused by SARS-CoV-2 is named as COVID-19 and has a very wide clinical spectrum [2]. The most frequent presentation requiring hospitalization is a bilateral pneumonia that in some patients may progress to respiratory and multiorgan failure [3]. Epidemiological features (elderly people, man, non-White people) and pre-existing conditions (comorbidities) are key determinants for a worse outcome of COVID-19 [4,5,6,7]. People with autoimmune diseases (AD) are also considered to be at increased risk of having a more severe infection [4], considering they have an underlying abnormal immune response and that are often under immunosuppressive therapy [8]. 

Sarcoidosis is a systemic granulomatous disease that affects adults older than 50 years-old in more than half the cases [9] with a slight predominance in women and an estimated prevalence of 6 cases per 10,000 people [10], that is classified from a pathogenic point of view as a T-cell mediated autoimmune disease [11], The most commonly involved organs are the lungs and regional lymph nodes, the skin, the liver and the eyes [12]. Sarcoidosis have some specific features that could favoring an increased risk for developing a severe COVID-19, including interstitial lung disease (in more than 50% of patients) and the damage of other internal organs leading to chronic failures (the kidneys and liver) [13]. An additional link between sarcoidosis and SARS-CoV-2 may be the key role of angiotensin converting enzyme (ACE) 2 as an essential receptor for the virus to enter the cell [14,15]. However, the studies that have analyzed the impact of SARS-CoV-2 infection in large cohorts of patients with sarcoidosis are limited and most are isolated reported cases or small series of cases (Supplementary Table S1). The three largest studies have substantial differences in design and case definitions, including a study based on the results of a self-reporting questionnaire distributed among members of associations of patients (116 cases, but without a specific definition of SARS-CoV-2 infection), a cross-sectional study in patients hospitalized due to COVID-19 (37 patients, all having a virological-proven diagnosis) and a nationwide hospital-based study (36 patients, all but two with a virological-proven diagnosis) [16,17,18].

The objective of this study was to analyze the clinical characteristics and outcomes of SARS-CoV-2 infection in patients with sarcoidosis and to identify the risk factors associated with a more complicated infection in one of the largest multicenter clinical cohorts of patients with sarcoidosis from Southern Europe.

## 2. Methods

The SARCOGEAS-Study Group was established in 2015 with the aim of collecting a large series of patients with sarcoidosis from Spanish hospitals with substantial experience in the management of systemic autoimmune diseases. Both incident and prevalent cases were included according to the fulfillment of the following criteria proposed by the American Thoracic Society/European Respiratory Society/World Association of Sarcoidosis and Other Granulomatous Disorders (WASOG) 1999 statement on sarcoidosis [19]:

(a) clinical or radiologic findings consistent with sarcoidosis, such as pulmonary disease, uveitis, mediastinal bilateral hilar lymphadenopathy (BHL), or erythema nodosum;
(b) tissue biopsy with histologic evidence of non-caseating granulomas;
(c) absence of other causes of granulomatous disease.

Since biopsy is usually not considered necessary for histological confirmation of a diagnosis in patients presenting with manifestations highly consistent with the disease (e.g., Lofgren’s syndrome or Heerfordt’s syndrome), or with an asymptomatic bilateral hilar lymphadenopathy, we allowed the inclusion of patients lacking the histopathological criteria (b) only if they presented the two other criteria (a and c) and, in addition, at least one of the following features suggestive of sarcoidosis: elevated serum angiotensin-converting enzyme, organ-specific abnormal uptake on gallium-67 citrate scintigraphy, elevated lymphocyte count or elevated CD4/CD8 ratio in bronchoalveolar lavage fluid or active extrathoracic involvement classified as highly probable according to the WASOG extrathoracic classification [20,21]. 

The study was conducted in accordance with the amended Declaration of Helsinki. The Clinical Research Ethics Committee of the coordinating center (HCB2016/0181) approved the protocol and written informed consent was obtained from patients with current follow-up.

### 2.1. Design and Variables

By the first week of January 2021, all centers included in the SarcoGEAS Registry were contacted via email by MRC asking for patients included in the Registry diagnosed with SARS-CoV-2 infection according to the European Centre for Disease Prevention and Control (ECDC) recommendations [22] on the basis of the following features:

(a) Epidemiological link (having a close contact with a confirmed COVID-19 case in the 14 days prior to onset of symptoms);
(b) Clinical presentation (fever, cough, shortness of breath, sudden onset of anosmia, ageusia or dysgeusia, headache, chills, muscle pain, fatigue, vomiting and/or diarrhea);
(c) Microbiological confirmation (detection of SARS-CoV-2 nucleic acid in a clinical specimen and/or a positive result in serological tests).

We applied the classification of SARS-CoV-2 infection included in the ECDC recommendations:

1. Possible SARS-CoV-2 infection (any person meeting the clinical criterion);
2. Probable SARS-CoV-2 infection (any person meeting the epidemiological and clinical criteria);
3. Confirmed SARS-CoV-2 infection (any person meeting the microbiological criteria).

We defined as inclusion criteria a probable or confirmed SARS-CoV-2 infection (defined according to the EDCD recommendations) and as exclusion criteria, a possible SARS-CoV-2 infection (according to the ECDC recommendations), lack of information about epidemiological/clinical criteria and concomitant pulmonary infections diagnosed at the time of SARS-CoV-2 infection.

Data about SARS-CoV-2 infection was extracted from electronic health records by using a standardized de-identified form including demographics, comorbidities (chronic cardiovascular, pulmonary, kidney or hepatic diseases, active neoplasia), symptoms at the time of diagnosis of SARS-CoV-2 infection (symptomatic cases were classified as COVID-19), pharmacological treatment and clinical outcomes (including need for hospitalization/supplemental oxygen, intensive care admission, non-invasive or mechanical ventilation and death). Laboratory results were collected as close to the time of SARS-CoV-2 diagnosis or initial hospital admission as possible. 

Variables related to sarcoidosis were defined according to previous definitions [23], including extrathoracic involvement (defined according to the 2014 WASOG organ assessment instrument of clinical scenarios classified as highly probable or at least probable [21]); first-line therapeutic management (glucocorticoids, immunosuppressive agents and/or biological agents); and Scadding radiographic stages, that were evaluated in all cases with available chest X-ray at diagnosis and were defined as stage 0 (normal), stage I (bilateral hilar lymphadenopathy BHL without pulmonary infiltrates PI), stage II (BHL plus PI), stage III (PI without BHL) and stage IV (extensive fibrosis with distortion or bullae) [24]. The results of pulmonary function testing, measured within 2 months and 2 years before the time of SARS CoV 2 infection (or before the time of the last visit in those without infection), were also extracted for analysis in those patients with pulmonary involvement (stages ≥ II); a moderate/severe impairment in pulmonary function was defined as values of FVC < 70% of LLN and/or DLCO < 60% of LLN [18].

### 2.2. Statistical Analysis 

Descriptive data are presented as mean and standard deviation (SD) for continuous variables and numbers and percentages (%) for categorical variables. The Chi-square test was used to study the main features related to SARS-Cov-2 infection according to infection management (requirement for hospital admission). The t-test was used to compare quantitative variables. Logistic regression models adjusting for age and sex (as the key prognostic markers for a more complicated infection) were built to confirm the unadjusted univariate results; adjusted odds ratios (OR) and their 95% confidence intervals (CI) were calculated. In addition, a case-control study was carried out between patients with and without SARS-Cov-2 infection about differences in phenotypic expression of sarcoidosis, selecting among the entire cohort a 2:1 age-sex-matched control population without SARS-Cov-2 infection (negative microbiological results including detection of SARS-CoV-2 nucleic acid in a clinical specimen and/or a positive result in serological tests). All significance tests were two-tailed and values of *p* < 0.05 were considered significant. Statistical analyses were performed using SPSS software ver. 23.0 (IBM, Armonk, NY, USA).

## 3. Results

We evaluated 48 cases for inclusion in the study. A total of 45 patients fulfilled the inclusion criteria (43 were classified as definite cases and 2 as probable cases; the remaining 3 cases were classified as possible cases and were excluded). In all cases but one, sarcoidosis was diagnosed before SARS-CoV-2 infection. There were 28 women (62%) and 17 men (38%) with a mean age of 55.4 years (range 31 to 89) (Table 1). There were 3 main epidemiological clusters of transmission comprising family, work (mainly in healthcare facilities) and unknown transmission. Baseline comorbidities were reported in 23 (54%) patients, mainly hypertension, diabetes mellitus, cardiovascular diseases and chronic pulmonary, kidney and liver diseases. Thirty-six (80%) patients presented with at least one symptom suggestive of SARS-CoV-2 infection and were classified as COVID-19 and the remaining nine (20%) were classified as asymptomatic infection. The most frequent symptoms of COVID-19 included cough (67%), fever (61%), fatigue (42%), myalgias (28%) and dyspnea (22%). According to the microbiological studies, 43 (96%) were classified as confirmed infections (positive PCR result in 33, positive antigen tests in four and positive serological studies in six) and the remaining two (4%) were classified as probable infections, according to the ECDC definitions. 

**Figure 1:**
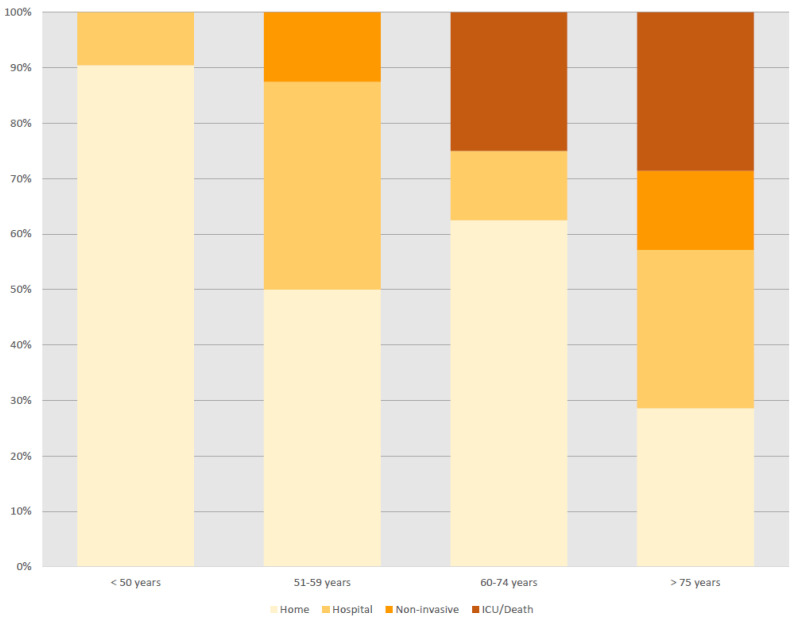
Individual outcomes of the 45 patients with sarcoidosis ordered from the youngest to the older age at diagnosis of SARS-CoV-2 infection.

SARS-CoV-2 infection was managed at home in 31 (69%) patients (with a close follow-up by GPs or by hospital at home programs) and at hospital in the remaining 14 (31%). In 22 patients, results from laboratory studies included raised values of CRP (73%), ferritin (59%), D-dimer (52%) and liver enzymes (48%) and lymphopenia (52%) as the most frequent abnormalities (Table 1). Chest radiographs were carried out in 20 patients and showed pulmonary infiltrates in 16 (80%). Specific treatment for SARS-CoV-2 infection was administered in 18 patients, including glucocorticoids in eight, hydroxychloroquine in 6, antiviral agents in 6six azithromycin in six, tocilizumab in two and anakinra in one patient. Among the 14 patients who were hospitalized, supplemental oxygen was required in 12 (86%), non-invasive ventilation in three (21%); two (14%) patients required admission to the intensive care unit and one (7%) mechanical ventilation. The main complications detected during hospital admission included superimposed bacterial infections in two cases and pulmonary embolism in one. Four (9%) patients died 5–10 days due to progressive respiratory failure related to bilateral pneumonia. Figure 1 summarizes the outcomes of the 45 patients with sarcoidosis and SARS-CoV-2 infection stratified by age subsets, showing increased rates of hospitalization/poor outcomes in older patients. According to the number of patients with sarcoidosis included by the participating centers (*n* = 878), the estimated frequency of SARS-CoV-2 infection was 5.1%.

Stratification according to the management of SARS-CoV-2 infection (requirement or not of hospital admission) showed that patients who required hospitalization had an older mean age (64.9 vs. 51.0 years, *p* = 0.006) and a higher frequency of baseline comorbidities (79% vs. 39%, *p* = 0.023), including a higher frequency of cardiovascular disease (64% vs. 23%, *p* = 0.016), diabetes mellitus (43% vs. 13%, *p* = 0.049) and chronic liver/kidney diseases (36% vs. 0%, *p* = 0.002) in comparison with those who were managed at home. Patients who required hospital admission also showed a statistically-significant trend for a higher frequency of active disease under immunosuppressive therapy (glucocorticoids and/or immunosuppressive agents) at the time of SARS-CoV-2 diagnosis (57% vs. 29%, *p* = 0.072). With respect to COVID-19 symptomatology, patients who required hospitalization presented more frequently with fever (79% vs. 35%, *p* = 0.011) and dyspnea (50% vs. 3%, *p* = 0.001). In patients with pulmonary involvement (stages II and III), a baseline moderate/severe pulmonary impairment in function tests was associated with a higher rate of hospitalization, although the difference was not statistically significant (50% vs. 23%, *p* = 0.219). Age- and sex-adjusted multivariate analysis identified the age at diagnosis of SARS-Cov-2 infection as the only independent variable associated with hospitalization (adjusted OR 1.18, 95% CI 1.04–1.35) (Table 2). 

With respect to the main features related to sarcoidosis phenotype, there were no statistically-significant differences for the main epidemiological, clinical, radiological, laboratory and histological characteristics of sarcoidosis between age-sex-matched patients with and without SARS-CoV-2 infection, except for a higher frequency of pulmonary involvement (stages II and III) in those with SARS-CoV-2 infection (64% vs. 46%, *p* = 0.045, OR 2.17, CI 95% 1.04–4.53). However, baseline pulmonary function tests showed a similar rate of moderate/severe impairment in patients with and without SARS-CoV-2 infection (Table 3).

## 4. Discussion

In this study, we have tried to capture the broadest, real-life spectrum of SARS-CoV-2 infection in patients with sarcoidosis, including not only hospitalized cases, but also asymptomatic patients and those diagnosed with a mild COVID-19 that were followed up at home in a primary care setting or under hospital at home programs. We have estimated a frequency of SARS-CoV-2 infection of 5.1%, a similar figure than that reported for the Spanish population (5%) [25]. The prevalence of SARS-CoV-2 infection in patients with sarcoidosis has been estimated in only one US study that identified five (2.1%) patients with SARS-CoV-2 infection out of 238 sarcoidosis patients during the first pandemic wave [26].

The clinical presentation of COVID-19 in our patients with sarcoidosis (signs and symptoms at presentation, laboratory results and radiographical abnormalities) was similar to that reported in the largest reported cohorts of infected patients [27,28], suggesting that individuals with sarcoidosis and SARS-CoV-2 infection could be managed with the standard of care that is being applied for the general population. With respect to the main outcomes of SARS-CoV-2 infection, we found that 31% of our patients with sarcoidosis required hospitalization and among them, 86% required supplemental oxygen, 14% required admission to ICU and 7% mechanical ventilation, with an overall mortality rate of 9%. These figures are similar to that reported in patients with primary Sjögren syndrome infected with SARS-Cov-2 [29] and in those with other autoimmune diseases, with an overall rate of 10% for ICU admission and a mortality rate of 12% [8]. 

The outcomes of SARS-CoV-2 infection in patients with sarcoidosis have been analyzed in several studies with a very heterogeneous design [16,17,18] (Supplementary Table S1). The frequency of hospital admission in our patients was very similar to that reported in the survey study [16] but clearly lower than that reported in the other two studies [17,18], reflecting probably that a substantial number of our patients were diagnosed in a primary care setting. We also found lower figures for poor outcomes (ICU admission, death) than that reported in previous studies. In comparison with previous studies, we have also included patients diagnosed during the second and third waves of pandemic in our country and this could explain the better prognosis of the infection in our patients, considering the worse outcome of COVID-19 reported in those who were hospitalized during the first wave of pandemic in comparison with subsequent waves [30,31].

We found that the main baseline features associated with a more complicated SARS-CoV-2 infection (age and chronic comorbidities) were similar to those identified in studies including general population [4] or patients with autoimmune diseases [8]. Hospital admission was more frequent in patients with sarcoidosis having baseline cardiovascular, kidney and/or liver chronic diseases and, especially, in those diagnosed with SARS-CoV-2 infection at an older age; the age was the unique risk factor independently associated with hospital admission in the multivariate adjusted model. Although we found that patients with a baseline moderate/severe pulmonary impairment in function tests had a higher rate of hospitalization, the difference was not statistically significant, probably due to the small number of patients with severe interstitial lung disease in our cohort, as has been reported in people with sarcoidosis from Southern Europe (CER). In the study by Morgenthau et al. [18] carried out in the US, the authors reported that in patients hospitalized due to COVID-19, a diagnosis of sarcoidosis was not associated with adverse outcomes except in those patients with moderately/severely impaired pulmonary function [18]. Several characteristics of the study by Morgenthau et al. [18] may explain why the study found a statistically significant association, including that the study was exclusively focused on hospitalized patients and that the US cohort has substantial epidemiological and clinical differences in comparison with our cohort (65% African American people vs. <5% and pulmonary involvement in 95% vs. 64%, respectively). Ethnicity plays a key role in the disease severity and mortality of sarcoidosis and African American people have the highest frequency of the more severe Scadding pulmonary stages (stages III–IV in 30%) and have an increased hospitalization rate and a 12-fold higher age-adjusted mortality rate in comparison with White people [32].

In our study, we found a statistically significant trend for a higher risk of requiring hospital admission due to COVID-19 in patients with sarcoidosis with active disease under immunosuppressive therapies. Probably, the small number of patients who required hospital admission in our series (linked to the lower disease severity of the disease in people from Southern Europe) contributed to give a result which may not be sufficiently powered to detect a difference between the groups. In fact, data from the International Registry [33] reported that 18 (44%) out of 41 patients with sarcoidosis included in the registry were receiving corticosteroids and there were no significant differences in hospitalization rates compared with those who were not, while another study showed that African American patients with chronic sarcoidosis treated with disease-modifying anti-rheumatic drugs (DMARDs) or anti-tumor necrosis factor (TNF) therapy did not have an increased risk of respiratory or life-threatening complications of COVID-19 in comparison with the general population [26].

In only one of our cases, SARS-CoV-2 infection was coincident with the disease diagnosis suggesting a potential sarcoidosis triggered by the virus. There is only one additional case reported by Behbahani et al. [34] in a patient who developed a cutaneous sarcoid involvement two weeks after developing COVID-19 pneumonia. Some studies have reported that mediastinal lymphadenopathies can be associated with COVID-19 pneumonia in 3–5% of patients [35,36]. In the largest retrospective studies, there is a wide variation in the frequency of mediastinal lymphadenopathies, with some studies reporting no cases [37] and others, a frequency of nearly half the cases [38]. Unfortunately, no information about the outcomes or histopathological studies supporting the presence of non-caseating granulomas was detailed in these patients. 

This study has a retrospective, observational design and therefore, the methodological limitations inherent to this design should be well acknowledged and explained. First, a selection bias cannot be discarded in our study, considering the great heterogeneity in the accessibility to evaluate the status of infection of all patients with sarcoidosis among the participating centers (including both symptomatic and asymptomatic cases), that may be very different even among regions of the same country. This approach was irretrievably associated with a lower degree of availability of medical examinations performed (laboratory and imaging studies) in those patients who were managed in a primary care setting. In addition, it should be considered that patients with chronic diseases may have an increased risk of being tested for SARS-Cov-2 [39,40], although this has not been reported specifically for patients with sarcoidosis. The retrospective approach to data collection also places limitations on the generalizability of our findings, not only by the small number of patients studied, but also by a specific disease phenotype linked to the predominant ethnic profile (a population overwhelmingly White from Southern Europe) that is characterized by a higher frequency of Löfgren syndrome and Scadding stage I together and lower frequency of severe pulmonary involvement [32,41]. Strengths of the study are the analysis of SARS-CoV-2 infection in one of the largest clinical registries of sarcoidosis and that all of our cases were resolved or had a known resolution status at the time of manuscript writing. 

In summary, sarcoidosis-infected individuals seemed to be similarly affected by SARS-CoV-2 infection compared with the general population in terms of clinical presentation. Notably, some presenting symptoms (fever and dyspnea), baseline comorbidities, active disease requiring immunosuppression and especially an older age at the time of infection were risk factors for a more complicated infection requiring hospital admission. The clinical phenotype of sarcoidosis was not related to with a poor outcome of SARS-CoV-2, although this lack of association could be linked with the low rate of moderate/severe pulmonary involvement in the studied cohort. These results underscore the need for a specific close monitoring of SARS-CoV-2 infection in elderly patients with sarcoidosis, especially in those with concomitant cardiopulmonary diseases and chronic liver or renal failure.

## Figures and Tables

**Table 1:** Main features of SARS-CoV-2 infection in 45 patients with sarcoidosis.

		Patients	Frequency
**Age, Years (Mean, Range)**	55.4 (31–89)	(*n*/N)	(%)
Sex	Male	17/45	37.8
	Female	28/45	62.2
Comorbidities	Any comorbidity	23/45	54.1
	Hypertension	15/45	33.3
	Diabetes mellitus	10/45	22.2
	Chronic pulmonary disease	8/45	17.8
	Cardiovascular disease	5/45	11.1
	Chronic renal/liver disease	5/45	11.1
Active therapies for sarcoidosis	Any	28/45	62.2
	Corticosteroids	15/45	33.3
	Immunosuppressant agents	7/45	15.6
	Biological therapies	0/45	0.0
Positive contact tracing	Family	16/45	35.6
	Work	8/45	17.8
	Not identified	21/45	46.7
SARS-CoV-2 infection	PCR	33/45	73.3
	Antigen tests	4/45	8.9
	Serological tests	6/45	13.3
	Clinical diagnosis	2/45	4.4
COVID-19 symptomatology	Symptomatic cases	36/45	80.0
	Cough	24/36	66.7
	Fever (temperature ≥37.5 °C)	22/36	61.1
	Fatigue	15/36	41.7
	Myalgias	10/36	27.8
	Dyspnoea	8/36	22.2
	Headache	6/36	16.7
	Arthralgias	5/36	13.9
	Ageusia/dysgeusia	4/36	11.1
	Gastrointestinal symptoms	4/36	11.1
	Anosmia	4/36	11.1
	Sore throat	2/36	5.6
	Thoracic pain	2/36	5.6
Respiratory features	Baseline O_2_ saturation		
	<95%	9/23	39.1
	<90%	3/23	13.0
	BPM > 20	7/23	30.4
Radiological features	No infiltrates	4/20	20.0
	Pulmonary infiltrates	16/20	80.0
Laboratory parameters	Hemoglobin value <12 g/L	6/22	27.3
	Platelets count <150,000/mm^3^	3/22	13.6
	Platelets count >450,000/mm^3^	2/22	9.1
	White cells count <4000/mm^3^	4/22	18.2
	White cells count >10,000/mm^3^	4/22	18.2
	Lymphocytes count <1000/mm^3^	11/21	52.4
	Raised D Dimer levels	11/21	52.4
	Raised LDH levels	9/21	42.9
	Raised ferritin levels	10/17	58.8
	Raised liver enzymes levels	10/21	47.6
	Raised CRP levels	16/22	72.7
COVID-19 treatment	Any specific therapy	18/45	40.0
	Thromboprofylaxis	9/45	20.0
	Glucocorticoids	8/45	17.8
	Hydroxychloroquine	6/45	13.3
	Azithromycin	6/45	13.3
	Antiviral agents	6/45	13.3
	Tocilizumab	2/45	4.4
	Anakinra	1/45	2.2
Management	Home	31/45	68.9
	Hospitalization	14/45	31.1
Duration of hospital stay, days (mean, range)	11 (2–33)		
Complications during admision	Respiratory failure (suppl O_2_)	12/14	85.7
	Bacterial infections	2/14	14.3
	Pulmonary embolism	1/14	7.1
	Intensive care unit admission	2/14	14.3
	Invasive mechanical ventilation	1/14	7.1
Outcomes	Death	4/45	8.9
	Recovery	41/45	91.1

**Table 2:** Demographic, clinical, radiological and laboratory features stratified by SARS-CoV-2 infection severity defined as requirement or not of hospital admission.

Clinical Variables	Managed at Home (*n* = 31)	Hospitalization (*n* = 14)	Bilateral *p*-Value
**Sex (men)**	10 (32.3)	7 (50)	0.326
**Age, years (mean +/− SD)**	51.03 ± 14.47	64.93 ± 16.27	0.006 *
**Ethnicity (white)**	22 (71.0)	10 (71.4)	1.000
**Comorbidities, any**	12 (38.7)	11 (78.6)	**0.023**
Cardiovascular (a)	7 (22.6)	9 (64.3)	**0.016**
Diabetes mellitus	4 (12.9)	6 (42.9)	**0.049**
Chronic liver/kidney failure	0 (0)	5 (35.7)	**0.002**
**Positive contact tracing**			0.898
Family	11 (35.5)	5 (35.7)	
Work	5 (16.1)	3 (21.4)	
Not identified	15 (48.4)	6 (42.9)	
**Baseline active therapies**			
Glucocorticoids	8 (25.8)	7 (50.0)	0.172
Immunosuppressive agents	3 (9.7)	4 (28.6)	0.180
Glucocorticods and/or immunosuppressive agents	9 (29.0)	8 (57.1)	0.072
Scadding radiological stages **			0.265
Stage 0	3 (9.7)	0 (0)	
Stage I	10 (32.3)	3 (21.4)	
Stage II	15 (48.4)	7 (50.0)	
Stage III	3 (9.7)	4 (28.6)	
Baseline pulmonary function tests ***			
Moderate/severe impairment	4/13 (30.7)	5/10 (50.0)	0.219
**COVID-19 symptomatology**			
Fever	11 (35.5)	11 (78.6)	**0.011**
Cough	16 (51.6)	8 (57.1)	0.759
Dyspnea	1 (3.2)	7 (50.0)	**0.001**
Gastrointestinal symptoms (b)	2 (6.5)	2 (14.3)	0.578
General symptoms (c)	13 (41.9)	6 (42.9)	1.000
Neurological features (d)	6 (19.4)	0 (0)	0.156
ENT symptoms (e)	4 (12.9)	2 (14.3)	1.000

* *p* = 0.013 in multivariate analysis, OR 1.18 (95 CI 1.04–1.35); (a) Cardiovasculare disease, HTA, heart failure; (b) Diarrhea, nausea, vomiting; (c) Fatigue, myalgias, arthralgias; (d) Headache; (e) Anosmia, dysgeusia, sore throat; ** No case classified as stage IV; *** Carried out in patients with pulmonary involvement (stages II and III). *p* values < 0.05 in bold

**Table 3:** Phenotype of sarcoidosis in patients with and without SARS-CoV-2 infection (1:2 age-sex-matched case control study).

Phenotype of Sarcoidosis	Sarcoidosis with SARS-CoV-2 Infection (*n* = 45)	Sarcoidosis without SARS-CoV-2 Infection *** (*n* = 90)	Bilateral *p*-Value
**Scadding radiological stages**			0.182
Stage 0	3 (6.7)	8 (8.9)	
Stage I	13 (28.9)	41 (45.6)	
Stage II	22 (48.9)	28 (31.1)	
Stage III	7 (15.6)	13 (14.4)	
Baseline pulmonary function tests *			0.762
Moderate/severe impairment	9/27 (33.3)	12/37 (32.4)	
**Clinical phenotypes**			
Thoracic involvement (stages I + II + III)	42 (93.3)	82 (91.1)	0.751
Pulmonary involvement (stages II + III)	29 (64.4)	41 (45.6)	0.045
Extrathoracic involvement	29 (64.4)	56 (62.2)	0.852
Number extrathoracic organs (mean, SD)	1.18 ± 1.25	1.28 ± 1.35	0.678
**WASOG extrathoracic involvement**			
Cutaneous	13 (28.9)	34 (37.8)	0.343
Lymph nodes	12 (26.7)	14 (15.6)	0.164
Ocular	1 (2.2)	6 (6.7)	0.663
Liver	7 (15.6)	12 (13.3)	0.795
Spleen	5 (11.1)	10 (11.1)	1.000
Salivary	0 (0)	5 (5.6)	0.169
ENT	0 (0)	2 (2.2)	0.552
Articular/bone	1 (2.2)	3 (3.3)	1.000
Neurological	0 (0)	7 (7.8)	0.095
Renal	5 (11.1)	6 (6.7)	0.505
Heart	0 (0)	1 (1.1)	1.000
**Diagnostic tests**			
Raised serum ACE levels	18/34 (52.9)	41/75 (54.7)	1.000
Raised serum calcium levels	6/32 (18.7)	9/76 (11.8)	0.370
Bx-proven dx	39 (86.7)	78 (86.7)	1.000
Sarcoidosis-related therapies **			
Glucocorticoids	18 (40.0)	52 (57.7)	0.051
Immunosuppressive agents	2 (4.4)	7 (7.8)	0.717
Biological agents	0 (0)	1 (1.1)	1.000

* Carried out in patients with pulmonary involvement (stages II and III); ** Therapies administered at any time after sarcoidosis diagnosis; *** Negative microbiological tests for SARS-CoV-2 infection.

## Data Availability

The data presented in this study are available on request from the corresponding author. The data are not publicly available due to Ethics policy.

## Supplementary Materials

The following are available online at https://www.mdpi.com/article/10.3390/v13061000/s1, Table S1. Previous studies reporting SARS-CoV-2 infection in patients with sarcoidosis.
