# Supplementary material for: Characterization and Outcomes of SARS-CoV-2 Infection in Patients with Sarcoidosis
Source: Viruses. 2021 May 27;13(6):1000. doi: 10.3390/v13061000 (PMC8228115; doi:10.3390/v13061000)
Supplement: Supplementary file 1 [file viruses-13-01000-s001.zip › viruses-1227529-supplementary.pdf]

viruses

Table S1. Previous studies reporting SARS-CoV-2 infection in patients with sarcoidosis.

First author	Country	Study design	Patients with sarcoidosis (n)	COVID-19 (n)	%	Hospital admission (n)	%2	ICU admission	%3	Death	%4	SARS-CoV-2 confirmed
Present study	Spain	Nationwide cohort	878	45	5,13	14	31,11	2	14,29	4	28,571	PCR in 43
Jeny et al	France	Nationwide cohort	nd	36	nd	28	77,78	13 of 28	46,43	5	17,857	PCR in 34
Morgenthau et al	US	Cross-sectional in COVID-19	nd	37	nd	22	59,46	9 of 22	40,91	6	27,273	PCR
Baughman et al	International	Survey in patiens from associations	nd	116	nd	18 of 114	15,79	6 of 18	33,33	nd	nd	Not defined
Gianfrancesco et al	International	Observational	nd	41	nd	nd	nd	nd	nd	nd	nd	PCR
Kiani et al	Iran	Observational	nd	10	nd	0	0,00	0	0,00	0	0	PCR
Mananasala et al	US	Observational	238	5	2,10	3	60,00	2 of 3	66,67	1	33,333	PCR
Ramdani et al	Morocco	Case report	nd	1	nd	1	na	0	na	0	na	PCR
Opoca et al	Poland	Case report	nd	1	nd	1	na	1	na	0	na	PCR
Padala et al	US	Case report	nd	1	nd	1	na	1	na	0	na	PCR
Bénezit et al	France	Case report	nd	1	nd	1	na	0	na	0	na	PCR
Gyorfi et al	Germany	Case report	nd	1	nd	1	na	0	na	0	na	PCR
Ng et al	US	Case report	nd	1	nd	1	na	1	na	1	na	PCR
